# A static glucose-stimulated insulin secretion (sGSIS) assay that is significantly predictive of time to diabetes reversal in the human islet bioassay

**DOI:** 10.1136/bmjdrc-2023-003897

**Published:** 2024-03-14

**Authors:** Ruth Damaris Molano, Antonello Pileggi, Hubert M Tse, Cherie L Stabler, Christopher A Fraker

**Affiliations:** 1 Cell Transplant Center, Diabetes Research Institute, University of Miami, Coral Gables, Florida, USA; 2 Microbiology, Molecular Genetics, and Immunology, University of Kansas Medical Center, Kansas City, Kansas, USA; 3 Department of Biomedical Engineering, University of Miami, Coral Gables, Florida, USA; 4 J Crayton Pruitt Family Department of Biomedical Engineering, University of Florida Herbert Wertheim College of Engineering, Gainesville, Florida, USA; 5 Diabetes Research Institute, University of Miami Miller School of Medicine, Miami, Florida, USA

**Keywords:** Islets of Langerhans Transplantation, Insulin Secretion

## Abstract

**Introduction:**

Static incubation (static glucose-stimulated insulin secretion, sGSIS) is a measure of islet secretory function. The Stimulation Index (SI; insulin produced in high glucose/insulin produced in low glucose) is currently used as a product release criterion of islet transplant potency.

**Research design and methods:**

Our hypothesis was that the Delta, insulin secreted in high glucose minus insulin secreted in low glucose, would be more predictive. To evaluate this hypothesis, sGSIS was performed on 32 consecutive human islet preparations, immobilizing the islets in a slurry of Sepharose beads to minimize mechanical perturbation. Simultaneous full-mass subrenal capsular transplants were performed in chemically induced diabetic immunodeficient mice. Logistic regression analysis was used to determine optimal cut-points for diabetes reversal time and the Fisher Exact Test was used to assess the ability of the Delta and the SI to accurately classify transplant outcomes. Receiver operating characteristic curve analysis was performed on cut-point grouped data, assessing the predictive power and optimal cut-point for each sGSIS potency metric. Finally, standard Kaplan-Meier-type survival analysis was conducted.

**Results:**

In the case of the sGSIS the Delta provided a superior islet potency metric relative to the SI.

**Conclusions**

The sGSIS Delta value is predicitive of time to diabetes reversal in the full mass human islet transplant bioassay.

**WHAT IS ALREADY KNOWN ON THIS TOPIC**

- The static glucose-stimulated insulin secretion (sGSIS) is a useful islet potency metric implemented in islet product release criteria.

**WHAT THIS STUDY ADDS**

- The Delta as a metric increases sensitivity and specificity of the sGSIS in predicting time to diabetes reversal in the full-mass transplant bioassay.

**HOW THIS STUDY MIGHT AFFECT RESEARCH, PRACTICE OR POLICY**

- This method is a rapid and accurate potency assay discriminating between good and suboptimal islet preparations. This could be useful in clinical transplant screening preventing the use of suboptimal organs.

## Introduction

The last decades have seen substantial advances in islet cell and stem cell-derived beta (SC-β) cell transplantation as a potential curative therapy for the treatment of type 1 diabetes mellitus. One persistent obstacle to ensuring that optimal cellular products are used for clinical procedures is the need for reproducible potency assessment(s).^1 2^ Historically, product release criteria for clinical islet transplantation rely on the Stimulation Index (SI; insulin produced in high glucose divided by the insulin produced in low glucose) obtained from glucose-stimulated insulin secretion (GSIS), in addition to dithizone zinc dye staining of the clusters (to assess purity of the cell product), islet cell viability (exclusion of DNA-binding dyes), and sterility.

An accepted ‘gold standard’ criterion for assessing human islet function, concomitant renal subcapsular transplant in chemically induced diabetic immunodeficient mice (aka in vivo bioassay), is not ideal since the results are not available until long after the cells have been transplanted into the human recipient.^3 4^ The data are often difficult to correlate with the human recipient outcome which is confounded by immune responses, islet equivalent (IEQ) number and purity (ie, non-endocrine tissue clusters in the graft) of the graft, and, finally, the number of donor pancreata used for transplantation. Despite preparation variability, there has been demonstrated correlation to clinical transplant outcome when single donors are used.^5^ With these technical limitations aside, it is accepted that if the islets can reverse hyperglycemia in mice, they will function with a degree of similarity in human recipients, barring immune rejection. Thus, the chemically induced diabetic immunodeficient mouse transplant bioassay, although retrospective, remains the potency metric of choice. Along with the bioassay, other assays, including, but not limited to, static GSIS (sGSIS), dynamic perifusion, oxygen consumption rate (OCR), single cell compositional assays and purine ratios (ATP/ADP, ATP/ATP+ADP+P_i_), have been compared in single or multiparametric correlative studies with some success.^1 3 4 6–14^ However, the search for a quick, simple and predictive pretransplant in vitro assay continues.

Two more recently proposed in vitro assays for the assessment of islet function are the measurement of OCR and the revisited dynamic perifusion.^6 11 13–15^ OCR has demonstrated significant predictability in the hands of several groups but is limited by the complexity of the measurement systems and the need for specialized training of the operator. Additionally, it has only been used as a binary yes/no diabetes reversal assay overlooking subtle differences in potency related to diabetes reversal time. Based on our own OCR work and this study examining insulin secretion, potency is a more complex question.^6^

Recent technological innovations and the advent of microfluidics have brought dynamic perifusion to the forefront of islet and SC-β functional assessment. These systems allow for high-throughput study of basic function and more complex investigations into the dynamic effects of drugs and secretagogues on islet multihormonal secretion patterns.^16–25^ Despite these advances, the systems are prone to technical and budgetary limitations. Disturbances in flow and other perturbations such as the presence of air bubbles can have profound effects on secretory response. The time and money associated with the maintenance of the devices coupled with the increased sample number make the assay cost prohibitive. Additionally, some systems (microfluidic) can assay only a few tissue clusters (islets, SC-β) from a preparation. Given the large number of islets in the human pancreas (estimated 10^5^–10^6^), the generally accepted practice is that assessments using less than 0.1% of the total population are minimally representative of a cell aggregate population that is typically heterogeneous in both size and cytoarchitecture. Still, as a tool for studying hormonal secretory response to external stimuli, the dynamic perifusion is invaluable, but to date, there has been no definitive comparative study to the transplant bioassay.

The static incubation, or GSIS (sGSIS) assay, has been implemented since the inception of islet research to quantify the insulin output from a representative aliquot of naked or encapsulated islets in both basal and elevated glucose concentrations. Methods have varied substantially over the years with discrete variations in the medium used, glucose concentrations, and the techniques for performing the assay. As well, the method has been a tool for islet research into potent secretagogues, drug screening, the complex physiology of insulin secretion and the effects of isolation, preservation and culture.^26–32^ Traditionally, results have been informative about islet function, but not consistently predictive of islet potency in vivo with a high degree of sensitivity and specificity. Much of the existing literature has focused on the SI, the ratio of insulin secreted with exposure to high glucose to the insulin secreted in the first hour of low-glucose exposure. We postulated that the Delta would be a superior metric of islet function relative to the SI as we observed that islet preparations would frequently produce low differential amounts of insulin per IEQ, but the SI would be high (proportional response).

Differences in individual protocols, particularly in buffer compositions and procedures, may contribute to a lack of observed predictive value of the SI. Insulin secretion has been shown to be affected by small changes in pH and osmotic/stretching force and, therefore, slight variations in buffer composition could adversely affect differential insulin output expected in the sGSIS.^33 34^ In this work, we developed an operator-friendly column-based method to perform static glucose-stimulated insulin release. A suspension of the islets in a Sepharose slurry was done to minimize mechanical perturbation of the islets and to prevent aspiration of islet particles, problems associated with other sGSIS methods. This method has been successfully adopted and used by groups for research functional assessment of rodent and human islets as well as SC-β.^35–37^ This assay provides an inexpensive and quick (<8 hours) method, from initiation to results, that could be implemented in islet or SC-β centers to obtain critical pretransplant potency information once a similar immunodeficient mouse bioassay correlation has been established, as will be presented.

## Materials and methods

### Islet isolation and culture

Human islets were isolated using a modified version of the Ricordi automated method and were allowed to recover for 24 hours at 37°C in conventional islet medium (Corning CMRL 1066 [-] phenol red, L-glutamine) before transplantation and sGSIS viability assessment. On the day of potency assessments and transplantation, IEQs were counted based on conventional dithizone staining using an inverted stereomicroscope with a graded reticle. From these counts, an aliquot was dedicated for the hand-picking of technical replicates of 50 (n=6 preparations) or 100 (n=25 preparations) similarly sized islets for the sGSIS assay. Thirty-two consecutive human islet isolations were used for this study.

### Glucose-stimulated insulin release

A modified Krebs buffer (KRB) with 26 mM sodium bicarbonate, 25 mM HEPES and 0.1% w/v bovine serum albumin and either 2.2 mM (low glucose) or 16.7 mM (high glucose) was prepared and warmed to 37°C in a standard 95% room air/5% CO_2_ incubator. Based on prior studies, the pH was titrated to 7.35–7.4 prior to use. Approximately 5 g of Sephadex G-10 DNA Grade (GE Healthcare), molecular weight cut-off of ~700 D, was added to a 50 mL beaker containing 20 mL of Dulbecco’s phosphate buffered saline without Ca^2+^, Mg^2+^ (Cellgro) and gently heated for 30 min to hydrate and swell the beads. Next, 10 mL Poly-Prep columns (Bio-Rad) were placed in Poly column rack (Bio-Rad) and 1 mL of modified KRB low-glucose buffer was added to each column. After the beads cooled, the slurry was added to the level of 400 µL in the graduated columns. The hand-picked islet aliquots were added and an additional 600 µL of bead slurry was added to each column to bring the final slurry volume to 1 mL. During this addition, the islets were mixed within the slurry to distribute them throughout the beads and prevent islet aggregation. Well packed, the void space of the bead slurry contains approximately 350 µL of liquid.

After the additional bead loading was complete, the bottom seals were removed from each column and an additional 4 mL of low-glucose buffer solution was added to each column to pack the beads and ensure that flow was unimpeded through each column. Additional bead slurry was added, if necessary, to maintain 1 mL packed bead volume. Flow in the columns ceased when the liquid level reached the surface of the beads keeping the fluid volume in each column constant and caps were placed on the column outlets to prevent leaking during incubation periods.

The sGSIS apparatus and prepared buffers were placed in the incubator for a 1-hour preincubation period. At the end of the first hour, 4 mL of fresh KRB low-glucose buffer solution was added to each column to wash out insulin secreted during the assay set-up and mechanical manipulation. The next 3 hours were sequential incubations with low-glucose solution, high glucose solution and a second low-glucose incubation. At the end of each of these hours, 1 mL of KRB low-glucose solution was added to each column, and the 1 mL eluate collected in an Eppendorf tube and immediately stored at −80°C for later insulin content assessment. Insulin was quantified using human insulin ELISA kits from Mercodia and/or by the Roche Cobas 6000 clinical chemistry analyzer according to the manufacturer’s protocol.

### In vivo assessment of islet function

Under protocols approved by the University of Miami Institutional Animal Care and Use Committee (A-3224-01), male athymic *nu/nu* (nude) mice were purchased from Envigo (formerly *Harlan Laboratories*, Indianapolis, Indiana) and housed in virus-free and antigen-free rooms in microisolated cages at the Division of Veterinary Resources of the University of Miami. In vivo studies were performed by the *DRI Preclinical Cell Processing and Translation Models Core*. Animals were rendered diabetic via a single intravenous administration of 200 mg/kg of Streptozotocin (Sigma-Aldrich, St Louis, Missouri). Non-fasting blood glucose was assessed by glucometer (Elite; Bayer, Tarrytown, New York) and mice with sustained hyperglycemia (>300 mg/dL) were designated for islet transplant. Grafts (2000 IEQs per recipient) were transplanted under the kidney capsule (range 1–7 per preparation) using recipients matched for glucose profile and body weight similarities. Graft size was matched by counting and assessment of pellet volume. After transplantation, non-fasting blood glucose values were assessed daily for the first week and then three times a week following for up to 100 days. Endpoint of the study was reversal of diabetes, defined as the time (days) to achieve stable non-fasting blood glucose <200 mg/dL (confirmed on at least three consecutive days). In animals achieving and maintaining normoglycemia after transplantation, nephrectomy of the graft-bearing kidney was performed to confirm return to hyperglycemia and exclude residual function of the native pancreas.

### Data analysis

The SI and the Delta (insulin produced in high glucose−insulin produced in low glucose 1) were examined for their predictability in determining time to diabetes reversal. Data were expressed as insulin output per hand-picked islet±SD. Animals that did not reverse were assigned a reversal time of 100 days, the in vivo monitoring endpoint, for analyses.

### Statistical analysis

General descriptive statistical analysis was performed on all sGSIS data. The high insulin output was compared with low 1 insulin output to assess if the values were statistically different. All statistical analyses (logistic regression, receiver operating characteristic (ROC), Kaplan-Meier survival, Mann-Whitney) were performed using GraphPad Prism for MacOS V.9.5.1.

### Simple logistic regression analysis

A series of simple logistic regressions were calculated. The ‘input’ data statistically analyzed were each of the metrics (SI and Delta). The objective of this analysis was to determine the optimal reversal time cut-points for the Delta and SI values that best organized the data sets (based on lowest p value plot) into two groups characterized by ‘rapid’ diabetes reversal or delayed/no diabetes reversal. In this method, the groups are assigned a binary classification, either 1 for rapid reversal or 0 for delayed/non-reversal. The assignment of 0 and 1 proceeds in a stepwise fashion along the reversal times, recording the p value at each step. The diabetes reversal time cut-point values are determined by the lowest p value associated with each logistic plot. This method is commonly used in success/failure analysis prior to ROC analysis to statistically determine the cut-points. As the arbitrary values assigned for non-reversal (100 days, in this case) are given a binary classification, the arbitrary value will have no impact on slope or related regression coefficients.^6^

### ROC and Kaplan-Meier survival analysis

ROC analysis was performed grouping the metric values (Delta or SI) using the optimal time to diabetes reversal cut-point determined for each by the simple logistic regression analysis. From this, the resultant metric value that gave the highest sensitivity and specificity was designated the cut-point value of each metric (Delta or SI), based on previously published methods.^38^ Kaplan-Meier survival analysis (eg, % normoglycemic recipients of human islet grafts) was then performed based on the grouping of metric data implementing the respective cut-points. Finally, a comparison between the two groups above and below the optimal metric cut-point for diabetes reversal time was performed using either the simple unpaired t-test or non-parametric Mann-Whitney U test, depending on results of data normality tests.

The calculated ROC area under the curve (AUC) is an indicator of predictive strength. In diagnostic tests, like the proposed potency metrics in this work, the range of AUC considered usually falls between 0.5 and 1. An AUC of 1 is indicative of 100% predictive ability, while 0.5 (line of unity) indicates that the metric has no ability to discriminate between positive or negative outcomes. To determine if the AUC measurements of the metrics were significantly different, the methods described by Hanley and McNeil for comparison of ROC curves from different algorithms/tests applied to the same data set were implemented.^39^

### Fisher’s exact test of diabetes reversal classification

To determine the ability of each metric to correctly classify diabetes reversal time, data were first grouped into a 2×2 table format with numbers of correctly and incorrectly classified diabetres reversal times (columns) for each metric (rows; Delta and SI). The Fisher’s exact test was performed on the data to determine the significance, if any, of one metric’s predictive power relative to the other.

## Results

### Donor characteristics

The donor data are included in the human islet checklists related to this manuscript. Of the donors, 13 were female and 19 were male. The mean donor age was 41.7±15.4 years with a range of 5–62 years. The mean body mass index was 28.5±5.6 with a range of 13.3–40.2. The mean cold ischemia time (CIT) was 11.8±4.8 hours with a range of 3.7–20.6 hours.

### Donor characteristics have no effect on assay outcome

**Figure 1:**
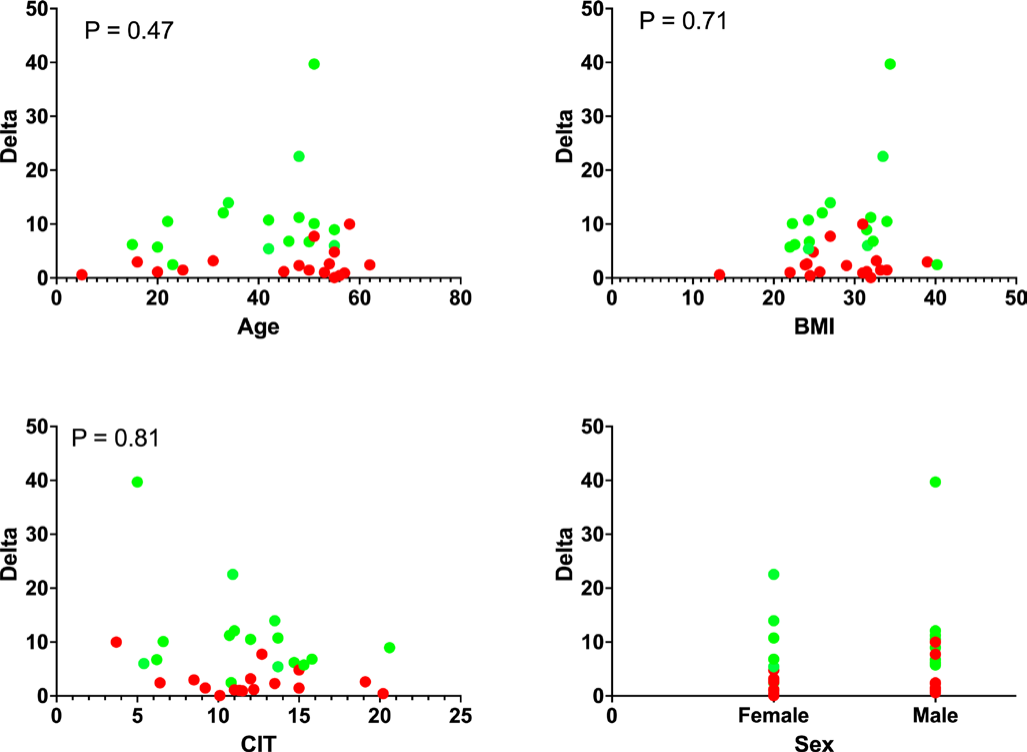
Grouping of measured static glucose-stimulated insulin secretion (sGSIS) Delta values by donor variables. Green dots represent potency values that resulted in rapid diabetes reversal (<5 days) and red dots represent those that resulted in delayed diabetes reversal (≥5 days). The distribution of Delta value was not correlated to any donor variable as can be seen from the nearly equivalent distribution of rapid and delayed diabetes reversal across the range of values on the X-axis. BMI, body mass index; CIT, cold ischemia time.

Of note, there was no significant correlation between any of the donor characteristics and the potency values (Delta or SI). Additionally, when grouped by potency outcome and time to diabetes reversal, there was no significant difference between the groups (rapid diabetes reversal time vs delayed diabetes reversal time) in any of the donor variables. Figure 1 shows the distribution of the Delta value in relation to all donor variables for rapid diabetes reversal (green dots) and delayed diabetes reversal (red dots). The distribution of donor variables was not significantly different between the two groups further supporting the lack of correlation between the donor variables and the Delta value.

### Sequence of potency assessment

**Figure 2:**
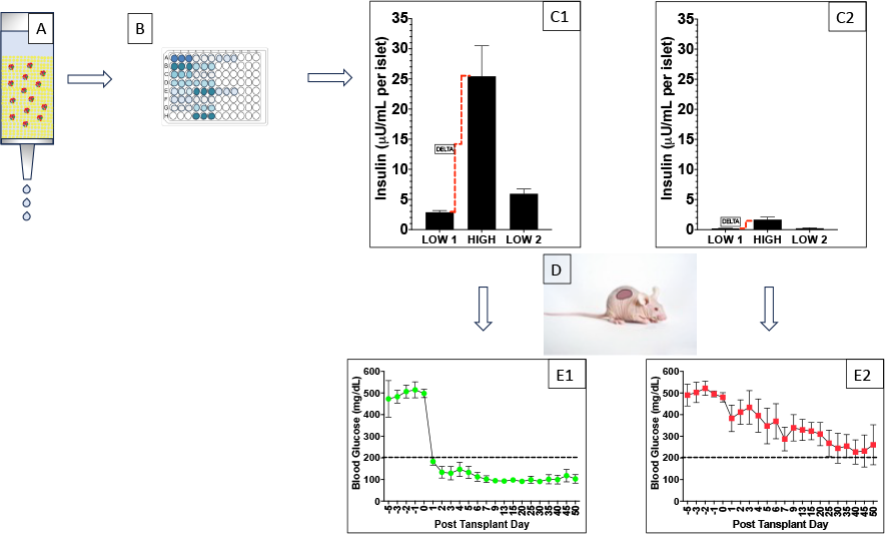
Work flow of the static glucose-stimulated insulin secretion (sGSIS) potency assay: islets were subjected to sGSIS loaded into the Sephadex bead slurry with a preincubation followed by three consecutive incubations/eluate collections in low, high and a second low-glucose exposure (A). Insulin content was measured by conventional ELISA (B), and the results analyzed and grouped through retrospective analyses into good (C1) or bad (C2) preparations. Islet transplants were performed in chemically induced diabetic immunodeficient mice for all preparations (D), and potency quantified as good (E1) or bad (E2) based on statistical analysis of time to diabetes reversal.

Figure 2 gives a schematic representation of the work flow for each human islet preparation studied in this manuscript. In figure 2A, islets are subjected to sGSIS loaded into the Sephadex bead slurry with a preincubation followed by three consecutive incubations/eluate collections in low, high and a second low-glucose exposure. Those samples were assayed for insulin content (figure 2B), and the results analyzed and based on retrospective analyses, classified as good (figure 2C1) or bad (figure 2C2) preparations. Full-mass subrenal capsular transplants were performed in chemically induced diabetic immunodeficient mice for all preparations (figure 2D), and follow-up of fasting glucose indicated either good (figure 2E1) or reduced (figure 2E2) potency.

### Summary of sGSIS data

Table 1 outlines the results of the sGSIS assay performed on 32 consecutive human islet preparations. Of the 32 preparations, 28 had significantly differential insulin response in the high glucose incubation relative to low 1. Of note, four of the five preparations where the insulin differential was non-significant still reversed hyperglycemia in the chemically induced diabetic immunodeficient mouse bioassay, although with longer cure times.

**Table 1:** sGSIS and transplant data for all 32 human pancreata used in potency assay development. Mean insulin, Delta and SI values±SD of the mean are provided along with number of transplants performed and the day of diabetes reversal

HP No	Islet/column	Low 1 insulin (µU/mL per islet)	High insulin (µU/mL per islet)	Low 2 insulin (µU/mL per islet)	High significantly different from low 1	Delta (µU/mL per islet)	Index	Transplant (n)	Reversal days
1	100	2.88±0.28	25.43±5.06	5.95±0.78	Y	22.55±4.89	8.8±1.4	7	1, 1, 1, 1, 1, 1, 1
2	100	2.17±0.39	5.14±0.65	2.22±0.38	Y	2.97±0.37	2.4±0.3	2	14, 24
3	100	3.54±0.07	10.25±1.82	3.72±0.78	Y	6.71±1.87	2.9±0.6	1	1
4	100	0.97±0.3	1.89±0.49	1.07±0.27	Y	0.93±0.19	1.9±0.1	2	6, 100
5	100	0.14±0.02	0.56±0.20	0.41±0.15	Y	0.42±0.19	3.9±1.1	1	12
6	100	0.14±0.03	0.73±0.1	0.37±0.06	Y	0.59±0.07	5.2±0.8	3	8, 11, 25
7	100	4.3±1.33	15.55±0.85	4.94±1.74	Y	11.24±0.6	3.8±0.9	1	1
8	100	3.89±0.35	6.36±0.54	4.14±2.44	Y	2.46±0.40	1.6±0.1	2	1,1
9	100	1.2±0.22	4.38±0.97	1.92±0.34	Y	3.17±1.16	3.8±1.5	2	92, 20
10	100	2.74±0.47	13.24±1.79	4.87±1.60	Y	10.49±2.1	5.0±1.4	6	1, 1, 1, 4, 4, 5
11	100	1.54±025	11.63±1.14	1.72±0.16	Y	10.09±1.33	7.8±2.0	2	1, 1
12	50	4.98±1.82	10.98±4.06	4.78±2.06	Y	6.00±1.16	2.2±0.2	2	2, 1
13	100	7.31±0.08	47.01±0.23	16.36±0.91	Y	39.7±0.15	6.4±0.1	2	1, 1
14	100	1.11±0.32	1.17±0.22	0.38±0.14	N	0.06±0.13	1.1±0.2	1	11
15	100	0.75±0.13	12.85±3.12	3.89±1.17	Y	12.09±2.99	16.9±1.5	1	1
16	100	2.46±0.72	3.49±1.35	1.09±0.5	N	1.03±0.63	1.4±0.1	1	9
17	100	0.73±0.49	1.83±0.2	0.39±0.03	N	1.1±0.28	3.1±1.8	1	14
18	50	0.96±0.22	7.16±0.98	2.48±0.82	Y	6.2±0.4	7.6±1.0	2	1, 2
19	50	9.34±0.38	18.28±1.7	7.84±2.26	Y	8.96±0.67	2.0±0.1	1	2
20	100	1.26±0.14	6.08±0.2	1.42±0.03	Y	4.82±0.34	4.9±0.7	1	100
21	50	9.54±3.0	23.5±6.04	14.38±1.64	Y	13.96±1.58	2.5±0.1	1	4
22	50	1.84±1.28	3.02±2.30	1.66±1.26	N	1.18±0.53	1.6±0.2	1	20
23	100	0.49±0.08	5.90±0.7	1.42±0.26	Y	5.41±0.66	12.1±1.4	1	1
24	100	1.40±0.26	12.14±2.06	3.40±0.63	Y	10.74±1.07	8.9±2.4	1	4
25	100	0.98±0.17	3.28±1.0	1.50±0.19	Y	2.3±0.91	3.3±0.8	1	6
26	100	1.32±0.29	11.31±2.11		Y	9.98±1.96	8.7±1.7	1	100
27	100	0.2±0.03	1.66±0.43	0.25±0.06	Y	1.46±0.45	8.6±3.3	1	100
28	100	1.96±0.29	4.39±1.04	1.89±0.34	Y	2.43±0.76	2.2±0.2	1	100
29	100	0.92±0.52	2.37±1.10	1.81±0.55	N	1.45±0.63	2.7±0.7	1	100
30	100	2.14±0.93	8.96±3.94	7.37±3.44	Y	6.82±2,91	4.2±0.3	2	2,2
31	50	0.92±0.14	6.64±0.80	0.98±0.08	Y	5.72±0.90	7.4±1.8	1	2
32	100	0.81±0.23	3.43±0.85	0.95±0.08	Y	2.62±0.64	4.3±0.3	1	100
33	100	2.37±0.39	10.10±1.35	2.57±0.15	Y	7.73±1.22	4.3±0.7	1	8

Mean insulin, Delta and Stimulation Index (SI) values±SD of the mean are provided along with number of transplants performed and the day of diabetes reversal.

sGSIS, static glucose-stimulated insulin secretion.

## Data analysis

### Simple logistic regression analysis

**Figure 3:**
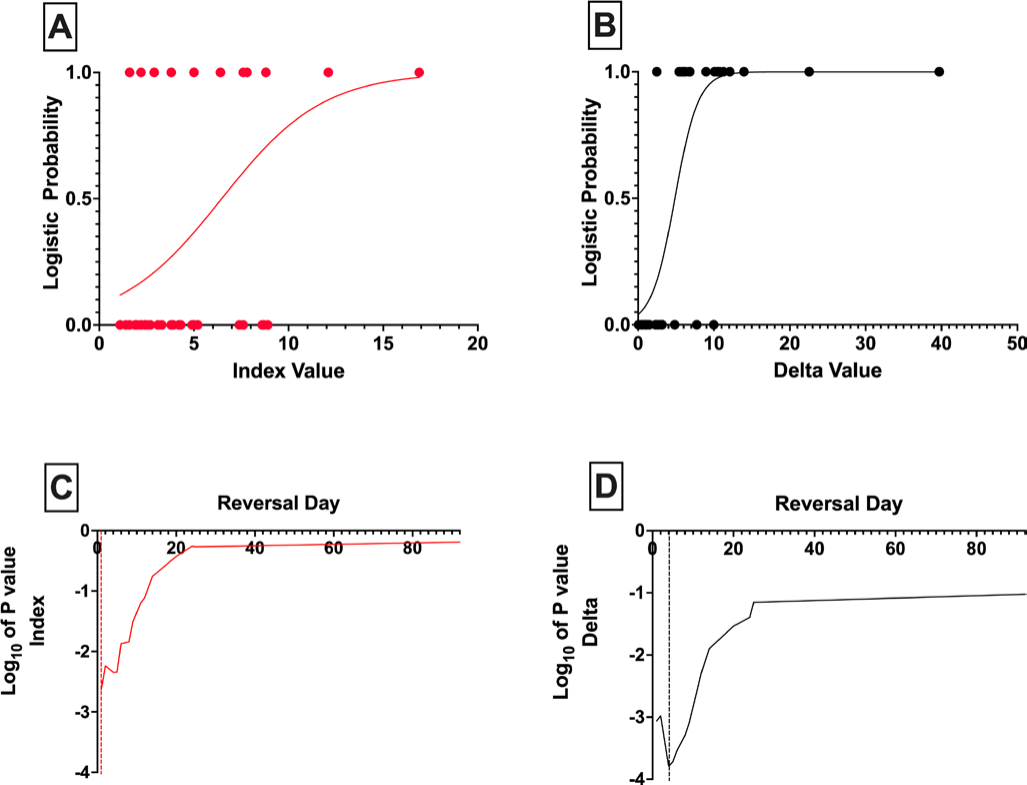
Final logistic regression plots for the Index (A) and the Delta (B) based on the recursive p value plot determination of the ideal cut-points for each metric (C for the Index and D for the Delta). The dashed lines in (C) and (D) indicate the diabetes reversal time cut-points determined by the statistical analysis.

For the purposes of comparing the two presented potency metrics (SI and Delta) and determining the optimal diabetes reversal time cut-point, a simple logistic regression analysis was performed. Based on the statistical analysis, the optimal diabetes reversal time was grouped into ≤5 days or >5 days for the Delta and ≤1 day or >1 day for the SI. Two statistical values were used to assess cut-point, goodness of fit by Tjur’s R^2^ and the analysis slope p value. For the Delta, the optimal Tjur’s R^2^ was 0.65 and optimal p value was 1.9×10^−4^. For the SI, the optimal Tjur’s R^2^ was 0.22 and optimal p value was 2.3×10^−3^. Figure 3 shows the p value plot of the logistic regression analysis used to determine the diabetes reversal day cut-off for survival analysis.

### ROC analysis metric cut-point determination

**Figure 4:**
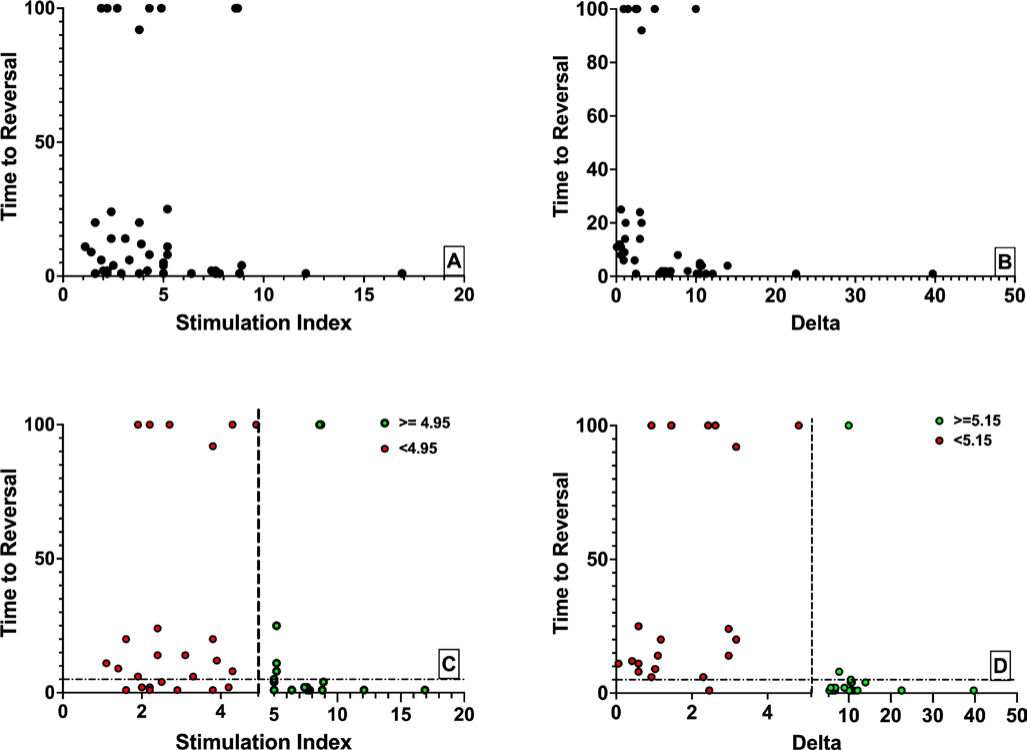
Raw data for the Stimulation Index (SI) (A) and Delta (B) values versus time to diabetes reversal in transplanted chemically induced diabetic immunodeficient mice in potency assessment of 32 consecutive human islet preparations. The same data grouped according to receiver operating characteristic (ROC) analysis determined the cut-off points for both the SI (C) and the Delta (D).

The optimal metric cut-point values determined by ROC analysis were 5.15 µU/mL per islet and 4.95 for the Delta and SI, respectively. These values were implemented to statistically group the data for survival analysis. Figure 4A,B shows the raw data for the SI and the Delta, respectively. Figure 4C,D shows the same data grouped using the cut-point values as dashed vertical lines. Here, green circles represent experimental replicates with values above the cut-point, and red below the cut-point. From the plots in figure 4C,D, it is clear that the number of both false positive (lower left quadrant) and false negative (upper right quadrant) classifications is reduced using the Delta relative to the SI, visually suggestive of its superiority as a metric.

### ROC analysis

**Figure 5:**
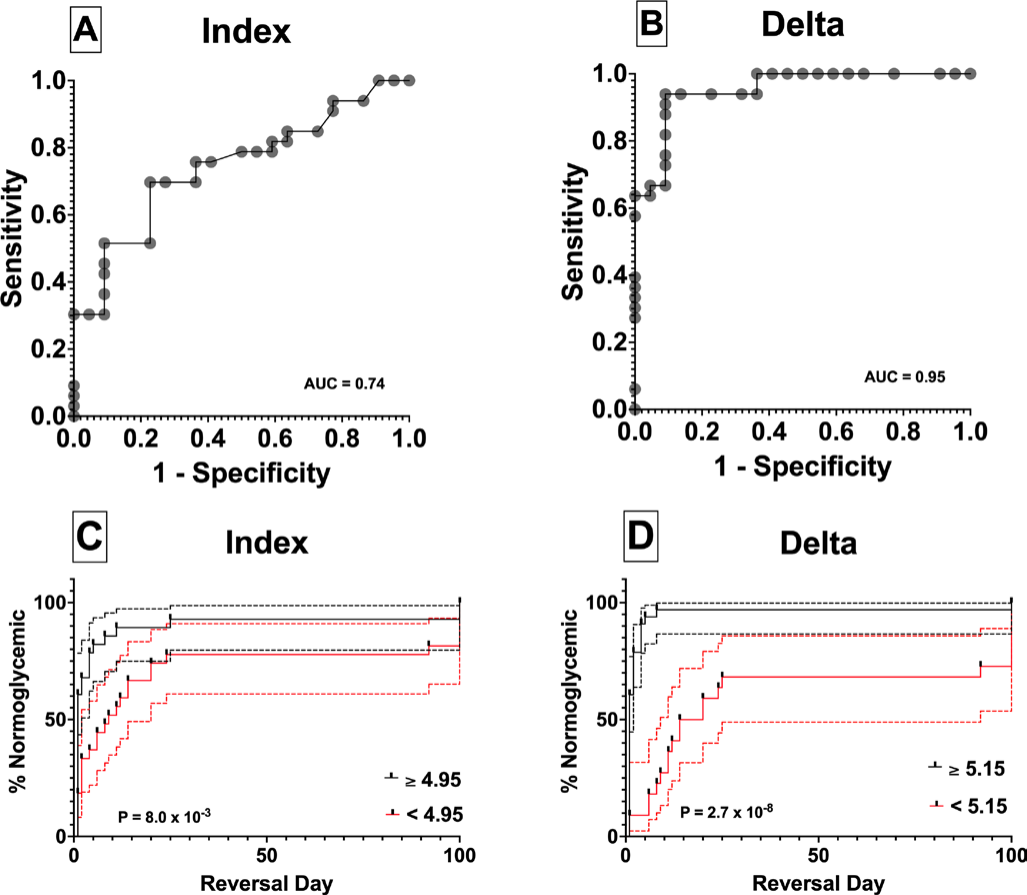
(A, B) Receiver operating characteristic (ROC) curves for the Stimulation Index (SI) (left) and the Delta (right). The analysis demonstrates greater area under the curve (AUC) and higher sensitivity and specificity for the Delta relative to the SI confirming the superiority of the Delta analysis as a predictive metric of time to diabetes reversal of hyperglycemia in the chemically induced diabetic immunodeficient mouse bioassay. (C, D) Kaplan-Meier survival analysis of chemically induced diabetic immunodeficient mouse bioassay transplants grouped based on the cut-off points of each respective potency metric, SI (left) and the Delta (right). For each graph, the solid black line represents islet preparations with potency values greater than or equal to the cut-off point, and the solid red line represents preparations with potency values less than the cut-off point. The dashed lines represent the 95% CIs for each plot.

Figure 5A,B shows the results of the ROC and survival analysis for each metric. The Delta was the most predictive metric with an ROC AUC of 0.95, indicative of excellent predictive ability. The optimal sensitivity and specificity of the Delta were 90.9% and 93.9%, respectively. The index was significantly less predictive with an ROC AUC of 0.74 and corresponding optimal sensitivity and specificity of 63.0% and 82.1%, respectively.

Figure 5C,D shows the results of Kaplan-Meier survival analysis based on the grouping of metric data implementing respective cut-points. The top curve (solid black line) in each plot represents the percentage of normoglycemic animals in the group above the cut-point of each metric while the lower curve (solid red line) represents those in the group below the cut-point. The dashed lines about each curve represent the 95% CIs for the percentage normoglycemic. The AUC of the ROC analysis along with the statistical comparison of the calculated AUC (p=0.003) confirmed that the Delta was the best metric for discriminating between islets that are likely to affect a rapid restoration of normoglycemia and those that may be delayed in restoring normoglycemia or fail to do so. Using the Delta, the ‘cure’ rate was 97% in the group above the cut-point and 73% in the group below the cut-point. The median diabetes reversal times were 1 and 17 days, respectively. For the index, the ‘cure’ rate was 93% in the group above the cut-point and 81% in the group below the cut-point. The median diabetes reversal times were 1 and 9 days, respectively.

### Fisher’s exact test of diabetes reversal classification

The Delta was significantly better than the SI in the correct classification of diabetes reversal times (p=0.004). While the SI accurately classified 37 of the 55 diabetes reversal times (62.2%), the accuracy of the Delta was superior (50 out of 55, 90.9%).

### Mann-Whitney U test of diabetes reversal groupings

**Figure 6:**
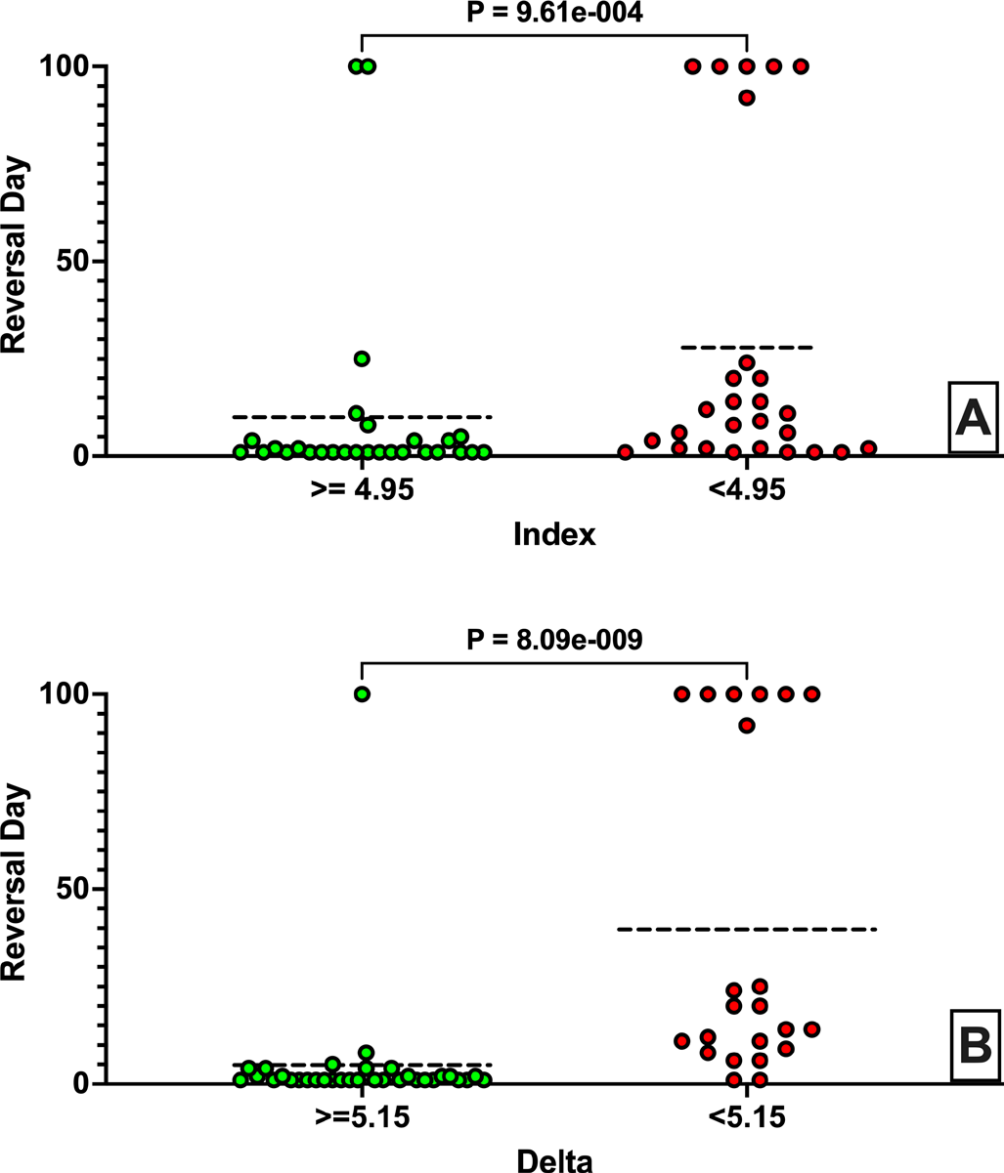
Diabetes reversal time groupings based on receiver operating characteristic (ROC) analysis for each metric with Mann-Whitney statistical analysis of differences between the two groups. The statistical grouping of both metrics resulted in significantly different mean times to diabetes reversal. The dashed line in each plot represents the mean diabetes reversal time. A depicts the grouping of the Stimulation Index values while B the grouping of the Delta values.

Figure 6 shows the diabetes reversal time (Y-axis) grouped according to cut-point for all metrics. The Delta grouping had a p value of 8.1×10^−9^ comparing diabetes reversal times above and below the metric cut-point. Mean diabetes reversal times±2×SEM were 4.9±5.9 days in the group above the cut-point and 39.7±17.8 days equal to or below the cut-point.

The index grouping had a p value of 9.6×10^−4^ comparing diabetes reversal times above and below the metric cut-point. Mean diabetes reversal times±2×SEM were 10.1±9.8 days in the group above the cut-point and 27.9±15.0 days equal to or below the cut-point. It is clear from this graph that SIs both above and below the cut-point result in similar diabetes reversal times more frequently than with the Delta.

**Figure 7:**
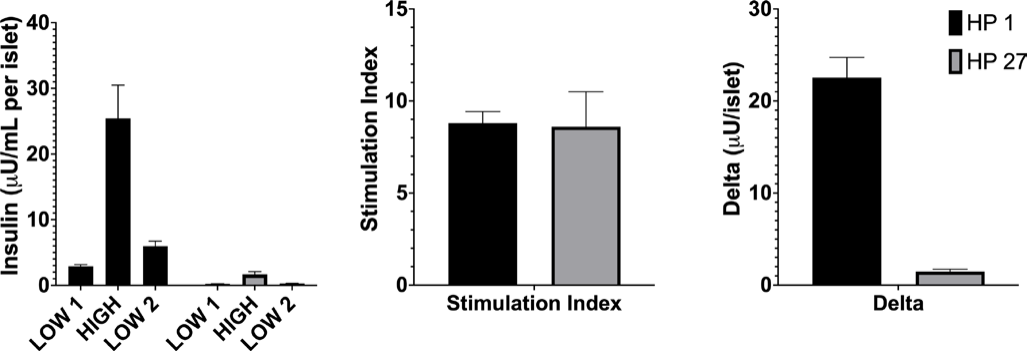
Representative comparison of human pancreata with similar Stimulation Index (SI) values but significantly different Delta values and transplant outcome. The Delta value was predictive of diabetes reversal/non-reversal in both HP while the SI, nearly identical in both preps, failed to distinguish transplant outcome differences. All seven mice transplanted with islets from HP 1 reverted diabetes at day 1, while the animal transplanted with islets from HP 26 failed to reverse diabetes.

Taken together, all of the presented analysis supports our hypothesis that while the SI is a significantly predictive metric, it is inferior to the Delta value. Human islets with low overall insulin output are still capable of having a significantly large SI while their total insulin output is not sufficient to regulate blood glucose and restore normoglycemia in chemically induced diabetic immunodeficient mouse recipients with a full-mass transplant. This is shown in figure 7 as an example.

All seven mice transplanted with islets from human pancreas 1 reverted diabetes at day 1 while the animal transplanted with islets from HP 26 failed to reverse. The mean stimulation indices were 8.8±1.4 and 8.6±3.3, not significantly different (p=0.91). The Delta values were 25.43±5.06 and 1.46±0.45, respectively. The Delta values were significantly different (p=5.7×10^−4^).

## Discussion

The index (SI) is a ratiometric representation of the differential insulin secretion in response to changing glucose levels. Historically used as a potency metric in correlative studies with in vivo graft function, it has been moderately predictive, at best. Low SI values are generally accurate predictors of graft failure or delayed function, but in our work and that of others, higher SI values are variable in their predictive capacity. In this study, we observed human islet preparations that had significantly high stimulation indices with low insulin output that failed to reverse hyperglycemia in thechemically induced diabetic immunodeficient mouse bioassay, and conversely, preparations with lower indices but greater insulin output (Delta) that reversed hyperglycemia almost immediately after transplant.

We posited that the amount of insulin produced in response to differential glucose concentrations was more predictive of graft success than the response ratio. The Delta, therefore, seemed a better metric in terms of in vivo potency. Much like patient insulin doses are scaled based on glycemic output of foods (eg, more insulin dosed with higher carbohydrate intake), logic dictates that the Delta insulin produced by cells in response to glucose is a better measure of function.

From our prior work examining OCR and from other published studies, the choice of incubation medium can be important to glucose-responsive changes. KRB is a minimal salt solution lacking components critical to long-term maintenance of cell function and viability, such as vitamins and amino acids. Complete medium, on the other hand, has sufficient amino acids to drive alternative metabolic pathways for both oxygen consumption and insulin secretion. The literature suggests that KRB buffer is reliable for detecting glucose-responsive changes.^6 13 14 40^ Conversely, complete media may mask these changes.^41–43^ The nutrient deprivation, although short term, experienced by islets during the sGSIS likely induces oxidative stress similar to the stress experienced after transplant in the chemically induced diabetic immunodeficient mouse bioassay. Inadvertently, we feel that this buffer further exposes the differences between ‘good’ and ‘bad’ preparations. Therefore, we recommend that the sGSIS be performed using minimal buffers as opposed to complete media.

As a predictor of time to reversal of hyperglycemia in full-mass human islet transplants in chemically induced diabetic immune-compromised mouse models, the Delta insulin had superior sensitivity and specificity relative to both the index and total insulin output. An ROC AUC of 0.95 is indicative of an excellent diagnostic test with a high likelihood of (1) accurately distinguishing between graft success and failure and (2) avoiding both false positives and negatives. Using the chemically induced diabetic athymic nude or similarly immune-compromised mouse model is critical to accurate determination of graft potency to prevent confounding results due to host immune responses.^3 4^ Additionally, it is clear from this work and others that using potency metrics to distinguish a binary output of success or failure in transplant could be misleading. Rather, in the case of this work and our work examining OCR, in vivo potency was better characterized by the time to hyperglycemia reversal. Given all the potentially confounding variables related to the human transplant outcome (eg, immune responses, recipient characteristics, diabetes duration, glycemic control), using cells that result in rapid reversal characterized by large quantitative differences in insulin output seems a logical choice.

Transplantation of autologous human islets is considered a therapeutic option for the palliative treatment of chronic pain in people undergoing total pancreatectomy.^44 45^ Transplantation of human islets obtained from allogeneic, cadaveric pancreata has shown remarkable impact in restoring metabolic control in people with brittle type 1 diabetes worldwide.^46^ Moreover, with the advent of increasingly unlimited supplies of islets from xenogeneic sources (eg, porcine) and human SC-β having consistent and reproducible ‘donor’ characteristics, dimensions and cytoarchitecture, it will likely be less challenging to develop reliable pretransplant potency assays. The consistency of these donor tissues would also allow for the elimination of other obstacles to transplant success, such as the mass transfer limitations related to the heterogeneous size distribution of isolated islets. Important donor factors confounding the field of historical islet transplantation such as warm ischemia time/CIT and the need for multiple organs to obtain sufficient islet number will no longer be relevant to long-term engraftment. Now, more than ever, a rapid and simple assay, such as the sGSIS method proposed in this work, could be readily implemented as an SC-β/porcine islet release criterion with some initial correlative studies with the chemically induced diabetic immunodeficient mouse bioassay.

## Data Availability

Data are available upon reasonable request. All data presented in this manuscript will be publicly available in its presented form. More detailed data is available upon reasonable request.
